# Effectiveness of Multimodal Intervention to Improve Blood Culture Collection in a Tertiary Care Hospital

**DOI:** 10.7759/cureus.53941

**Published:** 2024-02-09

**Authors:** Isha Kumthekar, Tejashree Urs, Deepashree Rajashekar, Krishna Karthik

**Affiliations:** 1 Department of Microbiology, JSS Medical College, JSS Academy of Higher Education and Research, Mysore, IND

**Keywords:** healthcare workers, kap analysis, multimodal interventions, septecemia, blood cultures

## Abstract

Introduction and methods

Blood culturing has become one of the backbone investigations for septicemia, fever of unknown origin, etc. This study was conducted to test the effect of multimodal interventions on the practical skills of healthcare workers (HCWs), raise awareness regarding the importance of aseptic blood culture collection practices, and increase compliance with the specific steps to be followed. Hence, this current interventional study was aimed at comparing the rate of isolation of contaminants grown among the blood culture specimens, assessing the knowledge, attitude, and practice (KAP) of HCWs collecting the blood culture specimen on various aspects of sample collection, educating the nursing staff regarding blood sample collection using a structured, pre-formed checklist, and emphasizing best practices for blood culture collection. All of the study's objectives were successfully met within the time frame specified. Using a pre-formed checklist and a Google form for KAP analysis eased the calculation.

Results

On analysis, the blood culture contamination rate in the pre-interventional phase dropped drastically from 6.16% to 3.03% in the post-interventional phase. The educational sessions conducted are a paramount reason for the reduction in the contamination rate. The HCWs were the least compliant towards the eighth step in the checklist (regarding palpation of skin); however, that too increased from 66.93% and 64.51% to a whopping 82.25% and 83.06%, respectively, with a chi-square value of 0.03 and a p-value of 0.85 (not significant).

Conclusion

Implementation of interventional studies as an audit like this in tertiary care hospitals can result in a significant reduction in blood culture contamination rates and can also improve the compliance of HCWs with blood culture protocols. This, in turn, can overall improve the effectiveness of blood culture (BC) testing and reduce mortality and morbidity in tertiary care hospitals. Further research can be conducted to brainstorm more methods to increase the compliance of HCWs. Better monitoring strategies can also be set to ensure low contamination rates. Additionally, some other methods can be derived to locate the source of contamination within the hospital environment and thus eliminate it. Similar interventions can be conducted for a longer duration of time to further reduce the blood culture contamination rate below 3% (as per the recommendations).

## Introduction

Despite many advances in the healthcare system over the last decade, bloodstream infections (BSI) continue to remain a grueling conundrum responsible for high mortality and morbidity worldwide. Also, BSI is the major cause of mortality among patients admitted to intensive care units (ICUs) [1,2]. Hence, an accurate and rapid diagnosis of pathogens causing BSI can help in initiating appropriate antimicrobial therapy, which can improve the clinical outcome by reducing mortality and morbidity [2]. Blood cultures play an important role in the management of sepsis. Since BSI is a medical emergency, the application of accurate methodology in the collection of blood samples for culture is extremely crucial. Contaminated blood cultures can prove to be troublesome from a diagnostic point of view. Errors in blood culture (BC) collection may hamper the diagnosis and consequent treatment of the patient and may also increase the incidence of cross-contamination (if sterile practices are not implicated). Commonly grown contaminants include skin flora like Propionibacterium spp., CoNS (coagulase-negative staphylococci), alpha-hemolytic streptococci, and diphtheroids [2]. Therefore, it is of absolute importance that appropriate entities are well versed in the correct timing of BC collection, skin preparation, sample collection site and number, and the appropriate volume of blood. In other words, the quality of blood culture strongly depends upon the collection of the appropriate blood sample [2-4]. Contamination rates of less than 3% are universally accepted as per international standards [3,4]. In the last quarter's analysis, we found that the blood culture contamination rate in our institute is 12%, which is much above the accepted value. Many hospitals like ours do not have a trained phlebotomist for the collection of blood culture samples. Hence, the current study is aimed at determining the effectiveness of a multimodal intervention to improve blood culture collection in a tertiary care hospital situated in South India. This study will also aim to emphasize best practices like the collection of the specimen before starting the first dose of antimicrobial agent, adequate volume to be collected, site of collection of the sample (especially when patients are on the central and peripheral lines), transport of blood culture bottles to the laboratory, etc.

This interventional study will enable us to gauge the practical knowledge and skills of our healthcare workers (HCWs), figure out the discrepancies in the same, and therefore plan out an effective interventional method to correct their blood collection practices. We aspire to improve the quality of blood collection for culture to decrease the rate of growth of contaminants, thus allowing us to provide our patients with the best care possible. Hence, this current study aimed to compare the rate of isolation of contaminants grown among the blood culture specimens during the pre-and post-intervention period, to assess the knowledge, attitude, and practice (KAP) of HCW’s collecting the blood culture specimen on various aspects of sample collection during the pre-and post-intervention period, to educate the nursing staff regarding blood sample collection during the interventional phase of the study using a structured, pre-formed checklist, and to emphasize best practices for blood culture collection during the intervention period.

## Materials and methods

The present study is an interventional study carried out for two months (from July 25, 2022, to September 18, 2022) in the adult medicine intensive care unit (MICU) of JSS Hospital, Mysore, Karnataka, India. The period of study is divided into three weeks of pre-intervention, two weeks of intervention, and three weeks of post-intervention. A total of 124 blood culture collection procedures from adult medicine ICUs were observed and recorded through a structured checklist. The study population included nursing staff, medical interns, and postgraduates posted to the adult medicine ICU. All blood culture procedures performed in the adult medicine ICU during a specific time of the day were included in the study. The study was divided into a pre-interventional phase, an interventional phase, and a post-interventional phase.

Pre-intervention phase (three weeks)

A blood culture sample collection audit was conducted daily for 1.5 hours by the direct observation method using a pre-formed structured checklist (Appendix 1), which was adopted from the ANTT (antiseptic non-touch technique). A well-structured knowledge, attitude, and practice (KAP) questionnaire survey regarding blood sample collection for culture were collected from nursing staff, medical interns, and postgraduates posted in the adult medicine ICU, and the data was analyzed. This method provided insight into the knowledge and practical skills of the HCWs and allowed for the preparation of an accurate plan to carry out the intervention. At the end of this phase, the rate of contaminants grown along with the blood culture specimens was analyzed using the following formula:

Blood culture contamination (BCC) rate = Number of contaminated cultures / Total cultures made x 100

Intervention phase (two weeks)

By utilizing the results of direct observation and the KAP survey, a strategic plan comprising multimodal strategies was created. Training and educational sessions were conducted during this period under the guidance of the infection control team. The specific steps mentioned in the checklist were explained to the HCWs, along with their proper sequence. The disadvantages of inaccurate collection of blood culture samples were also stressed. Also, posters related to blood sample collection for culture (Appendix 2) were displayed as visual reminders in the adult medicine ICU and the general medicine ward. The sessions were conducted with educational and linguistic barriers in mind. Additionally, on-site corrections were made daily through one-on-one interaction.

Post-intervention phase (three weeks)

The blood culture sample collection audit was done similarly to the pre-intervention phase. The rate of contaminants growing among blood culture specimens during this period was calculated using the same formula and compared with the pre-intervention period. Knowledge, attitude, and practice (KAP) questionnaire survey regarding blood sample collection for culture were collected from nursing staff, medical interns, and postgraduates posted in the adult medicine ICU, and the data was analyzed and compared with the pre-intervention period. This has enabled us to gauge the impact of the multimodal interventional strategies on the HCWs. All the data generated was entered in MS Excel (Microsoft Corporation, Redmond, Washington, United States) and analyzed using SPSS software (IBM Corp., Armonk, New York, United States). The rate of contaminants growing during pre-and post-intervention phases was calculated using the McNemar test. A P-value less than 0.05 was considered statistically significant.

## Results

Blood culture sample collection was audited for six weeks, three weeks before the intervention, and three weeks after the intervention. Around 124 procedures were observed in the pre-intervention and post-intervention periods, respectively. Two samples from different sites were collected into two blood culture bottles as per the standard protocols. The audit was assessed in the pre-and post-interventional phases. Blood culture contamination rates were also determined during these phases. The contamination rates in the pre-and post-interventional phases were 6.16% and 3.03%, respectively. (Table 1). Overall, blood culture positivity in the pre-interventional phase was found to be 4.40% and 6.92% in the post-interventional phase, with a statistically significant reduction in contamination rates.

**Table 1:** Presenting the blood culture contamination rates in the pre-and post-intervention phases *p value <0.05 is considered to be statistically significant

	Total number of cultures	Contaminated cultures	Blood culture contamination rate	P-value
Pre-intervention	454	28	6.16%	0.023*
Post-intervention	462	14	3.30%	

It was observed that during the pre-interventional phase, 95.16% of the subjects performed hand hygiene before collecting the samples into two blood culture bottles, respectively, and 100% used sterile gloves, applied a tourniquet, palpated the vein, and marked the area. Of the subjects, 95.96% and 92.74% used 70% alcohol and rubbed the skin vigorously (5cm circle) before collecting blood in bottles one and two, respectively. Around 87.9% of the subjects waited for 30 seconds to allow the skin to dry completely, and 99.1% used chlorhexidine or povidone-iodine to disinfect the site concentrically inside out for bottles one and two. Around 85.48% and 83.87% of the subjects waited for two minutes to allow the skin to dry. It was observed that 66.93% and 64.51% of the subjects did not palpate skin again after disinfection for both bottles, and 100% of the subjects used an alcohol wipe to clean the bottle top for bottle one and 99.1% for bottle two. The same needle was used for blood collection and injection into the BACTEC bottle by 100% of the subjects. It was also observed that 88.7% of the subjects collected the ideal volume, i.e., 8-10 ml for adults and 1-3ml for pediatric or sterile fluid, into both bottles.

After the interventional phase and during the post-interventional phase, the percentage of adherence to following the steps showed a significant increase (Table 2).

**Table 2:** Presenting the observations while collecting blood for culture in the pre-and post-intervention phases

Steps	Pre-intervention		Post-intervention	
	Bottle 1	Bottle 2	Bottle 1	Bottle 2
1. Performed hand hygiene	95.16% (118)	95.16% (118)	97.58% (121)	97.58% (121)
2. Used sterile gloves	100% (124)	100% (124)	100% (124)	100% (124)
3. Applied tourniquet, palpated the vein and marked the area	100% (124)	100% (124)	100% (124)	100% (124)
4. Used 70% alcohol and rubbed the skin vigorously (5cm circle)	95.96% (119)	92.74% (115)	100% (124)	98.38% (122)
5. Waited for 30 sec (allow the skin to dry)	87.9% (109)	87.09% (108)	95.96% (119)	96.77% (120)
6. Used chlorhexidine /povidone I_2_ to disinfect site concentric inside out	99.1% (123)	99.1% (123)	100% (124)	100% (124)
7. Waited for two minutes (allow the skin to dry)	85.48% (106)	83.87% (104)	96.77% (120)	98.38% (122)
8. Did not palpate skin again after disinfection	66.93% (83)	64.51% (80)	82.25% (102)	83.06% (103)
9. Used an alcohol wipe to clean the bottle top	100% (124)	99.1% (123)	100% (124)	100% (124)
10. Used the same needle for blood collection and injecting into the Bactec bottle	100% (124)	100% (124)	100% (124)	100% (124)
11. Volume collected (Ideal: 8-10ml for adult, 1-3ml for pediatric/sterile fluid)	88.7% (110)	79.8% (99)	95.96% (119)	95.96% (119)

During the pre-and post-interventional phases, two components were adopted that aimed at assessing the knowledge of blood sample collection among healthcare workers and others to assess their attitude and practice of blood sample collection for culture. The KAP survey had 54 HCWs participate in it. In the pre-interventional phase, 64.81% were aware that sample collection has to be done from two different venipuncture sites, 57.4% knew the correct technique of sterilizing the site for drawing blood, 42.59% palpated skin after disinfection, and 48.14% were aware of the WHO-assigned "My 5 Moments of Hand Hygiene." Of the participants, 53.79% knew about the minimum duration for which hand rub has to be performed; 75.92% were aware of the volume of blood to be collected in adults and pediatric cases/sterile fluids; 74.07% were aware of using an alcohol wipe to clean the bottle cap; and 72.22% knew the appropriate time for the collection of blood for culture. The post-interventional phase showed a significant variation in the knowledge-based questionnaire provided to the HCWs (Table 3).

**Table 3:** Presenting the responses to the knowledge-based questions during the pre-and post-intervention phases

Knowledge-based questions	Correct responses	
	Pre-intervention	Post-intervention
Collection of samples from two different venipuncture sites	64.81% (35)	83.33% (45)
Correct technique of sterilizing the site for drawing blood	57.4% (31)	88.88% (48)
Palpation of skin after disinfection	42.59% (23)	66.66% (36)
WHO-assigned "My 5 Moments of Hand Hygiene"	48.14% (26)	77.77% (42)
Minimum duration for which hand rub has to be performed	53.7% (29)	70.37% (38)
Volume of blood to be collected in adults and pediatric cases/ sterile fluids	75.92% (41)	90.74% (49)
Usage of an alcohol wipe to clean the bottle cap	74.07% (40)	85.18% (46)
Appropriate time for collection of blood for culture	72.22% (39)	83.33% (45)

A questionnaire based on application and practice was provided to the HCWs as a part of the second component in the pre-and post-interventional phases, and it was observed that there is a significant change in the post-interventional phase compared to the pre-interventional phase (Table 4).

**Table 4:** Presenting the results of the application and practice-based questions during the pre-and post-interventional phases

AP1. Did you follow all the steps as per the SOP/poster for collecting the sample?	Pre-intervention	Post-intervention
Yes	81.5% (44)	92.59% (50)
Yes, partially	18.5% (10)	7.4% (4)
No	0	0
AP2. Which of the following aspects will help you to adhere to the correct method of blood sample collection?		
Visual reminders like posters	35.2% (19)	40.74% (22)
Observing seniors	16.7% (9)	12.96% (7)
Educational sessions	27.8% (15)	24.07% (13)
Provision of a pre-formed checklist	20.4% (11)	22.22% (12)
AP3. What makes you not perform hand hygiene when required?		
Inadequate availability of hand rub products	9.25% (5)	14.81% (8)
Forgetfulness	88.88% (48)	85.18% (46)
Lack of motivation	1.85% (1)	0
Allergy to hand rub product	0	0
AP4. Your diagnosis is usually dependent on the blood culture?		
Always	33.3% (18)	37.03% (20)
Sometimes	66.7% (36)	62.96% (34)
Never	0	0
AP5. Do you deliver the drawn blood into the culture bottle with the same needle used for venipuncture?		
Always	38.9% (21)	72.22% (39)
Sometimes	57.4% (31)	25.92% (14)
Never	3.2% (2)	1.85% (1)
AP5. Are the standard precautions followed by you before and after collecting the sample for blood culture?		
Always	59.3% (32)	75.92% (41)
Sometimes	40.7% (22)	24.07% (13)
Never	0	0

## Discussion

Bloodstream infections (BSIs) and septicemia are known to be significant causes of morbidity and mortality in a healthcare setting, and blood cultures play a vital role in their diagnosis [5]. Identification of the etiological agents and appropriate treatment plays a vital role in reducing mortality and morbidity [6]. Pre-analytical phase workflow, i.e., several factors such as the technique of specimen collection, volume of blood collected, number of BCs drawn, time of collection concerning antibiotic administration, and transport time of blood cultures by any process in the laboratory attribute to the quality of the final results and the outcome [7-11]. Healthcare workers working in tertiary care hospitals with huge patient loads and busy schedules can attribute this to the dereliction of the preanalytical workflow of blood culture tests. These will directly affect the outcome of the investigation, which will directly determine the treatment provided to the patients. Hence, a continuous monitoring system and education of the staff should exist to reduce such outcomes in a tertiary care hospital. Therefore, this study was carried out to develop a multimodal intervention and, subsequently, to evaluate its effectiveness in improving BC collection practices in a tertiary care hospital. According to the recommendations from the Clinical and Laboratory Standards Institute (CLSI), a blood culture contamination rate of less than 3% is acceptable [12]. As compared to the pre-(6.16%) and post-interventional phases (3.03%), the current study's contamination rates show a considerable decrease in the post-interventional phase. The current study involved a multimodal intervention, which included educational intervention and displaying standard protocols for blood sample collection for culture and sensitivity at the collection sites. This has majorly contributed to the significant reduction in contamination in the post-interventional phase. Concentrating on other groups like HCWs on rotational shifts, interns, resident doctors, and newly joined HCWs can even reduce the contamination further.

There are many reasons for blood culture contamination, like patients' own skin commensal flora, HCWs being the source, and sometimes from the hospital environment [13-16]. The major limitation of this study is that the source of blood culture contamination was not assessed. Further, the compliance with the blood culture sample collection protocol, knowledge, and attitude of HCWs were evaluated using a pre-formed questionnaire. Compared to the pre-intervention group, there was a significant improvement in the compliance of HCWs to draw BCs in pairs. Several studies have documented that the collection of paired or multiple BCs results in improvements in pathogen isolation and also helps in differentiating contaminants from pathogens, and the results of our study also showed similar findings. Similarly, their knowledge of blood sample collection and attitude toward and practice of blood sample collection for blood culture showed a significant change in the post-interventional phase when compared to the pre-interventional phase.

## Conclusions

For decades, blood cultures have been considered a potential tool for saving lives. However there are several problems during blood sample collection that in turn increase contamination rates and can directly affect the patients, for example: long hospital stays, increased diagnostic tests, prolonged antimicrobial exposure, and unnecessary usage of antibiotics. This can directly contribute to the morbidity and mortality of patients. Tertiary care hospitals with huge patient loads can attribute this to these parameters. Hence, we tried to implement a comprehensive multimodal interventional study that aimed at assessing blood culture contamination, assessing the knowledge, attitude, and practice of HCWs collecting the blood culture specimen on various aspects of sample collection during the pre-and post-intervention period, educating the nursing staff regarding blood sample collection during the interventional phase of the study, and using a structured, pre-formed checklist to emphasize best practices for blood culture collection during the intervention period. Through this, we found that implementing studies like these can result in a significant reduction of BC contamination rates and can also improve the compliance of HCWs with BC protocol. This, in turn, can overall improve the effectiveness of BC testing and reduce mortality and morbidity in tertiary care hospitals.

## Figures and Tables

**Figure 1:**
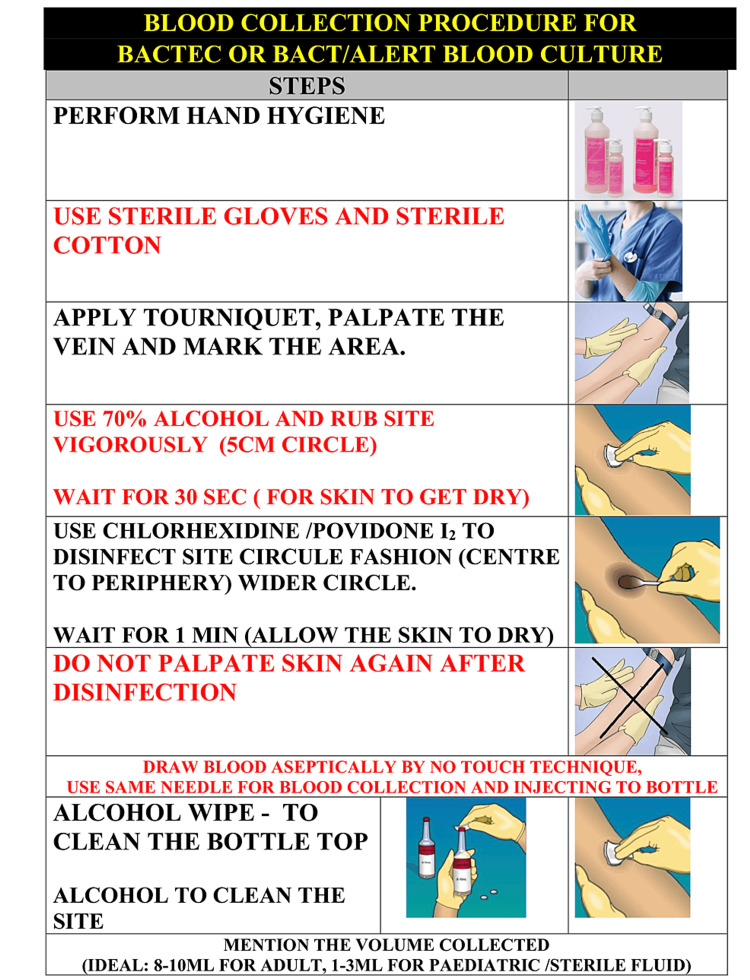
Blood culture collection poster

## Appendices

Appendix 1

**Table 5:** Checklist for blood sample collection

Checklist for blood sample collection		
Name: Age: Sex:		
Hospital No:		
Department: Unit:		
Date and time of blood culture sent		
Blood culture sent: a. Before starting antibiotics b. After starting antibiotics		
Provisional diagnosis:		
Site: CL/PL/V (Central line/Peripheral line/Venipuncture)	1st Bottle	2nd Bottle
1. Performed hand hygiene		
2. Used sterile gloves		
3. Applied tourniquet, palpated the vein and marked the area		
4. Used 70% alcohol and rubbed the skin vigorously (5cm circle)		
5. Waited for 30 sec (allow the skin to dry)		
6. Used chlorhexidine/povidone iodine to disinfect site concentric inside out		
7. Waited for two mins (allow the skin to dry)		
8. Did not palpate skin again after disinfection		
9. Used alcohol wipe to clean the bottle top		
10. Used the same needle for blood collection and injecting to Bactec bottle/blood culture bottle		
11. Adequate volume collected (Ideal: 8-10 ml for adults, 1-3 ml for Pediatric/ sterile fluids)

Appendix 2
